# Short-term survival of patients with advanced pancreatic cancer admitted to intensive care unit: a retrospective cohort study

**DOI:** 10.3332/ecancer.2022.1475

**Published:** 2022-11-23

**Authors:** Marina Junqueira de Almeida, Marcos Pedro Guedes Camandaroba, Antonio Paulo Nassar, Victor Hugo Fonseca de Jesus

**Affiliations:** 1 Medical Oncology Department, A.C. Camargo Cancer Center, Rua Prof. Antônio Prudente, 211, São Paulo, SP 01509-010, Brazil; 2 Intensive Care Medicine Department, A.C. Camargo Cancer Center, Rua Prof. Antônio Prudente, 211, São Paulo, SP 01509-010, Brazil; a https://orcid.org/0000-0003-4702-116X

**Keywords:** pancreatic, cancer, intensive, care, survival

## Abstract

**Background:**

Little is known about the outcomes of patients with advanced pancreatic cancer admitted to the intensive care unit (ICU) due to medical complications. We designed a study to evaluate their short-term (30-day) survival, predictors of short-term survival and chances of additional chemotherapy.

**Methods:**

We reviewed all patients with advanced (stage III or IV) pancreatic adenocarcinoma admitted to an ICU in a dedicated Brazilian cancer centre from 2009 to 2018 due to medical reasons. We fitted multivariate regression models to identify predictors of 30-day survival and additional systemic chemotherapy.

**Results:**

The study population consisted of 171 patients. Ninety-four patients (55.0%) had Eastern Cooperative Oncology Group (ECOG) performance status 2–4 and 146 (85.4%) had metastatic disease. Most patients (*N* = 75; 43.9%) were admitted to the ICU during first-line treatment. Median overall survival was 32 days (95% confidence interval (95% CI): 20–49). Survival rate at 30 days was 50.6%. ECOG performance status 2–4 was the only variable associated with lower probability of survival at 30 days in multivariate analysis (odds ratio: 0.28; 95% CI: 0.14–0.54; *p* < 0.001). Overall, 58 patients (33.9%) received additional chemotherapy and among all patients, 13.5% experienced clinical benefit from this treatment.

**Conclusion:**

Patients with advanced pancreatic cancer admitted to the ICU for medical reasons have a dismal prognosis. Early palliative care and refined tools to establish those who would benefit from an ICU trial could help improve patients’ care.

## Introduction

Pancreatic cancer currently represents the third most common cause of cancer-related death in the United States [1] and recent projections foresee increasing incidence and mortality rates in the next decade in both developed [2, 3] and developing countries [4]. In the advanced setting, the prognosis of the disease is ominous, with median survival as short as 4 months in population-based studies [5, 6].

However, survival outcomes have significantly improved with the widespread use of more active chemotherapy regimens [7, 8]. In parallel, the fraction of patients with pancreatic cancer admitted to intensive care units (ICUs) in North America has risen steadily [9], perhaps because of the perception that patients with clinical decompensation might benefit from these oncological treatments once their condition has stabilised.

Despite this trend, very little information is available regarding the outcomes of patients with advanced pancreatic cancer admitted to the ICU and putative harbingers of survival. In a large study from the Netherlands [10], patients with pancreatobiliary tumours had inferior survival when compared to those with other tumours, but most patients in this series were admitted to the ICU for post-operative care. Thus, more data on patients with advanced pancreatic cancer admitted to the ICU for clinical reasons are needed.

Thus, we designed a retrospective analysis of patients with advanced pancreatic cancer admitted to the ICU in our institution from 2009 to 2018. Our primary aim was to describe the survival outcomes of these patients. As secondary outcomes, we evaluated the frequency of clinical/radiological benefit of further oncological treatment and predictors of short-term survival and additional chemotherapy. Also, in an exploratory analysis, we aimed to devise a nomogram looking at the chances of 30-day survival according to the results of the regression model.

## Methods

This is a retrospective study carried out in a single large academic cancer-dedicated private hospital in São Paulo, Brazil. We used routinely collected data extracted from the electronic records of patients with advanced pancreatic cancer admitted to the ICU due to medical complications from 2009 to 2018. This study was approved by the Institutional Ethics Board Review, which waived the requirement for informed consent due to the retrospective and observational design of the study (number 2521/18N).

### Patients

We searched for patients admitted to the AC Camargo Cancer Center ICU using the Intensive Care Medicine Department database. We included patients aged 18 or more years-old, with pathologically confirmed diagnosis of stages III or IV (according to the 7th edition of the American Joint Committee on Cancer (AJCC) at the time of ICU admission) pancreatic adenocarcinoma, admitted to the ICU from 1 January 2008 to 31 December 2018 due to clinical complications. Patients were staged as stage III or IV based on pancreatic-specific abdominal CT and chest CT. Occasionally, patients would also undergo positron emission tomography/computed tomography (PET/CT)/CT and/or laparoscopy for staging purposes. We excluded patients with neuroendocrine tumours, those with concomitant malignancies and those admitted to the ICU to palliate symptoms or for post-procedural/post-operative monitoring. Only the first admission to the ICU due to medical reasons of a given patient was considered in this analysis.

### Variables

We collected data on age, sex, comorbidities, performance status within 30 days of the ICU admission (according to the Eastern Cooperative Oncology Group performance (ECOG) status scale), stage according to the 7th edition of the AJCC, sites of metastasis, setting of ICU admission (before treatment initiation, during first-line, second-line, third- or fourth-line or best supportive care (BSC)), reasons for ICU admission, date of ICU admission, date of ICU discharge, neutropenia at admission, use of organ-supportive therapies (vasoactive drug, haemodialysis and mechanical ventilation), additional systemic chemotherapy, clinical benefit from additional chemotherapy and radiological benefit from additional chemotherapy. For those patients with missing information on ECOG performance status, we inferred this variable from the description of the patient’s condition present at the last medical visit record before ICU admission. To improve confidence in this data, two investigators (MJA and MPGC) independently assessed this variable and in case of discordance, a review of the patient’s record by both physicians was undertaken to extract the most accurate performance status. These data were extracted from the AC Camargo Cancer Center ICU database and patients’ electronic records in the Medical Oncology Department.

### Outcomes

We defined overall survival as the time from the admission to the ICU to death from any cause or last follow-up; 30-day survival rate as the proportion of patients alive at 30 days. We defined clinical benefit as the proportion of patients with improved or controlled symptoms after additional systemic chemotherapy following discharge from the ICU among all included patients (according to the treating physician’s impression); and radiological benefit as the proportion of patients with radiological response (according to the treating physician’s impression and/or radiology report) after additional chemotherapy following discharge from the ICU among all included patients. Data on both clinical and radiological benefit were extracted by a senior medical oncologist (MPGC).

### Statistical analysis

We described the distributions of categorical variables using absolute frequencies and ratios and the distributions of numerical variables using medians and interquartile ranges (IQRs). The Kaplan–Meier estimator was used to calculate median survival times (and respective 95% confidence intervals (95% CIs)) and generate survival curves. Differences in time-to-event outcomes were assessed using the log-rank test. Median follow-up was calculated according to the inverse Kaplan–Meier method. We performed binary logistic regression models to identify putative factors associated with the survival status at 30 days (alive versus dead) and the receipt of additional systemic chemotherapy (yes versus no), using the following predefined variables: age (as continuous variable), sex (male versus female), ECOG (0–1 versus 2–4), age-adjusted Charlson comorbidity score (as continuous variable), stage (III versus IV) and treatment setting (before or during first-line versus during further lines of treatments or BSC). Data were reduced to comply with the ten events per degree of freedom rule-of-thumb [11]. In the regression model, we excluded the staging component of the Charlson comorbidity index to avoid collinearity with the variable stage. We handled missing data by performing complete-case analyses. We used all these variables to generate the multivariate model. Those variables with *p* values > 0.50 were excluded from the multivariate model in backward selection, as long as their removal did not result in meaningful changes in coefficients and their 95% CIs [12]. We assumed that there were no meaningful interactions between variables. We used the Tukey–Pregibon link test to look for model specification, the Hosmer–Lemeshow test to evaluate goodness-of-fitness, multivariate fractional polynomials to look for linearity of effect for numerical predictors and the scaled Brier score to explore the model’s overall performance. Calibration was assessed through calibration plots and discrimination was evaluated using the c statistic. We performed bootstrap with 200 draws to internally validate the findings of the 30-day survival logistic regression model. We also display optimism-corrected estimates of odds ratios (ORs) and 95% CIs according to the bootstrap method [13]. Model diagnostics are also portrayed after model adjustment by bootstrap shrinkage factor. Additionally, we devised a nomogram that predicts the 30-day survival probability using Stata’s nomology command and the final logistic regression model [14]. We also analysed survival according to the reason for ICU admission and need for organ-supportive therapies (vasoactive drug, haemodialysis and mechanical ventilation). These variables were not included in the logistic regression analyses to decrease the chances of overfitting. However, non-adjusted survival estimates according to these variables are presented separately. We considered a two-tailed *p*-value < 0.05 as statistically significant. All statistical analyses were performed using Stata (College Station, TX, USA) version 16.0.

## Results

**Figure 1.**
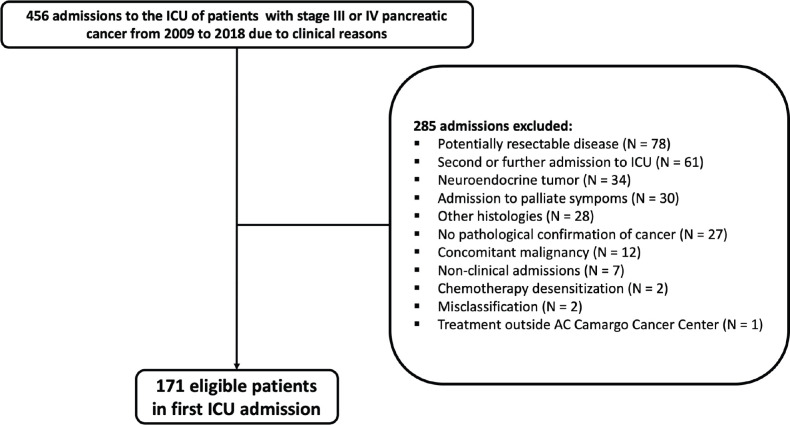
Study population flowchart.

Figure 1 depicts the patients’ selection process. Overall, the study population consisted of 171 patients. Median age was 66 (range: 39–87) years. A total of 94 patients (55.0%) presented ECOG performance status 2–4. The median age-adjusted Charlson comorbidity score was 9 (IQR: 7–10). Most patients had metastatic disease at ICU admission (*N* = 146; 85.4%) and, from these, 104 patients (71.2%) had hepatic metastasis. Most patients were admitted to the ICU during first-line treatment (*N* = 75; 43.9%) (Table 1).

### Reasons for ICU admission and characteristics of ICU stay

The most common reason for ICU admission was sepsis (*N* = 85; 49.7%) (Table 2). Thirty-nine patients (22.8%) had the diagnosis of cholangitis. Thirteen patients (7.6%) were neutropenic. The median ICU length-of-stay was of 4 days (IQR: 2–6). During the ICU stay, 69 patients (40.4%) received vasoactive drugs, five (2.9%) underwent renal replacement therapy and 31 (18.1%) received invasive mechanical ventilation. Overall, 75 patients (43.9%) receive either vasoactive drugs, haemodialysis or mechanical ventilation.

### Survival analysis

**Figure 2.**
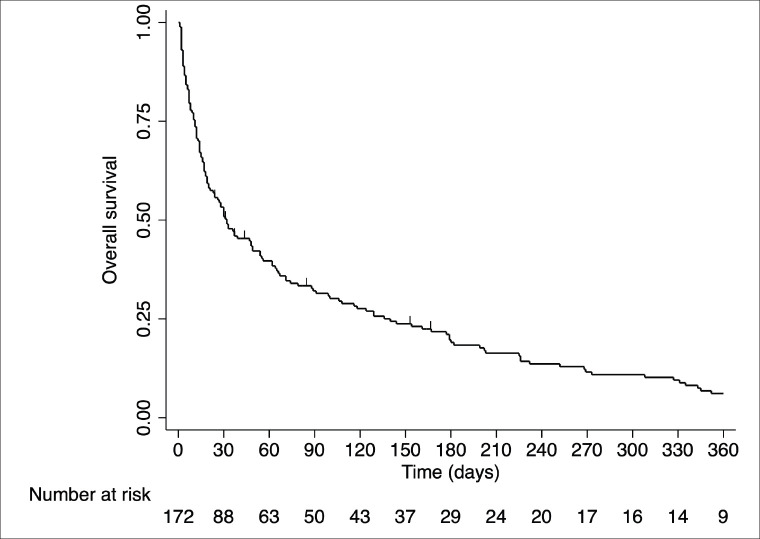
Overall survival from the time of ICU admission.

Median follow-up was 1,162 days (95% CI: 542–NA). Twelve patients were lost to follow-up. Overall, the median survival was 31 days (95% CI: 20–49) (Figure 2). According to the Kaplan–Meier estimator of the survival function, chances of being alive at 30, 90 and 180 days were 50.6, 32.3 and 19.2%, respectively. Third-nine (22.8%) patients died in the same ICU stay and 95 (55.6%) patients died in the same hospital stay. At 30 days, 79 (46.2%) patients had died. Five additional patients had unknown survival status at 30 days and, therefore, were excluded from the logistic regression model.

Supplementary Table 1 describes the overall survival according to the reason for ICU admission. There were no statistically significant differences in overall survival among the five main groups of reasons for ICU admission (*p* = 0.452). Supplementary Table 2 depicts the overall survival according to the use of specific organ-supportive therapies. Patients who underwent haemodialysis (median overall survival: 14 versus 33 days; *p* = 0.018) or mechanical ventilation (median overall survival: 11 versus 39 days; *p* < 0.001) experienced inferior survival outcomes.

**Supplementary Figure 1.**
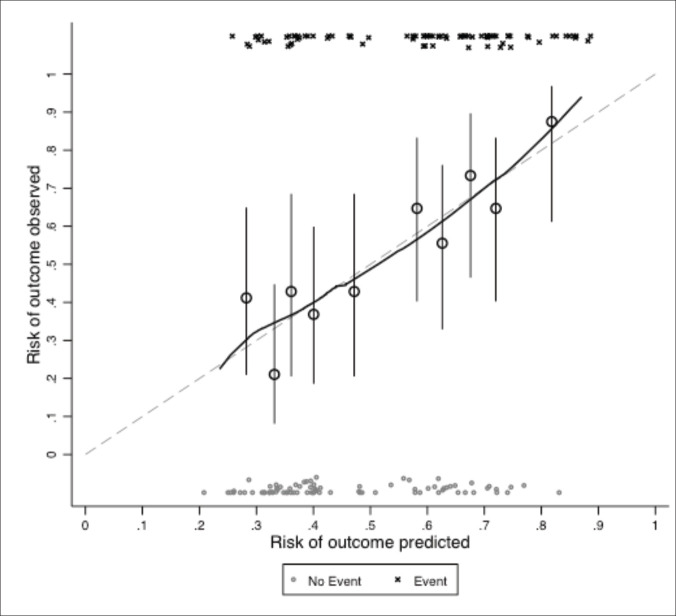
Calibration plot not adjusted for optimism.

**Supplementary Figure 2.**
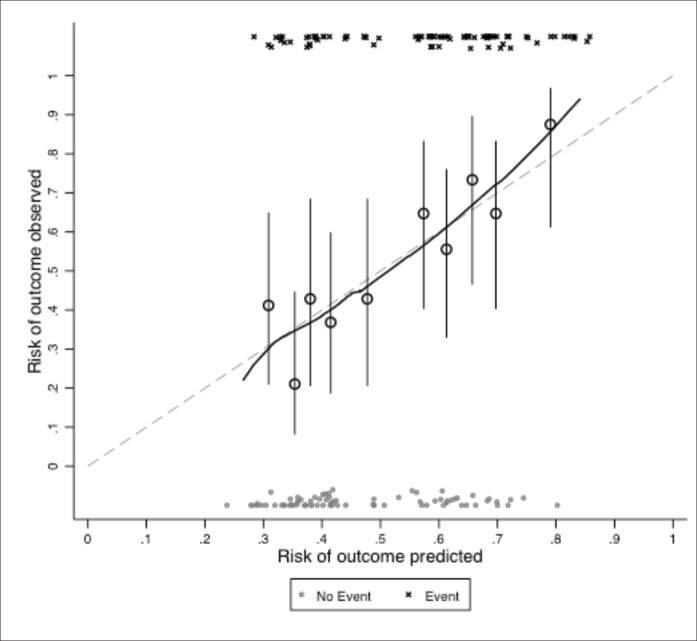
Calibration plot adjusted for optimism.

**Figure 3.**
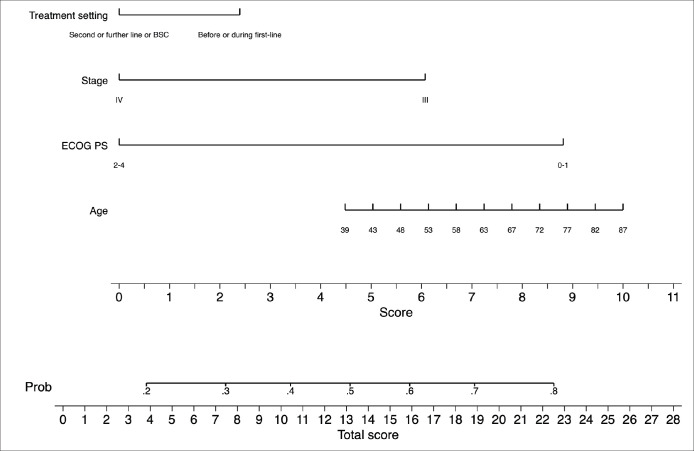
Nomogram predictive of survival status 30 days after ICU admission.

Table 3 portrays the final logistic regression model for 30-day survival. Sex and age-adjusted Charlson comorbidity score were excluded from the multivariate model as these variables proved to add very little to the model as described in the *Methods* section. In the multivariate analysis, only ECOG performance status 2–4 was independently associated with lower probability of being alive at day 30 (OR = 0.29; 95% CI: 0.15–0.56; *p* < 0.001). Model diagnostics (apparent and optimism-adjusted) are shown in Supplementary Table 3. Calibration plots (apparent and optimism-adjusted) are shown in Supplementary Figures 1 and 2. Using Stata’s nomology command, we then generated a nomogram based on age, ECOG performance status, stage and treatment setting to visually aid in the calculation of the survival probability at day 30 (Figure 3). The optimism-adjusted multivariable model is shown in Supplementary Table 4.

### Additional systemic chemotherapy

Fifty-eight patients (33.9%) underwent additional chemotherapy. Clinical benefit was perceived in 23 (13.5% of all patients). No patient experienced radiological benefit (response). In multivariate logistic regression analysis, ECOG 0–1 (OR = 3.45; 95% CI: 1.79–6.67; *p* < 0.001) was the only factor independently associated with higher chances of receiving additional systemic chemotherapy (Supplementary Table 5).

## Discussion

In this study, we show that only half of the patients with advanced pancreatic adenocarcinoma admitted to the ICU for medical reasons are alive at 30 days and that ECOG performance status 0–1 before admission was the only variable associated with improved survival. Additionally, 34% of the patients received additional chemotherapy after being discharged from the ICU; however, only 14% of all patients benefited from such treatment.

Recent data from the USA demonstrate that the proportion of patients with advanced pancreatic cancer admitted to the ICU has risen steadily from 1992 to 2006 [9]. A similar trend was seen in our study, with increasing numbers of patients admitted to the ICU more recently and a relatively constant number of new patients with pancreatic cancer per year in the study period [15]. Even though it is possible that in a cancer-dedicated hospital, patients are more likely to receive intensive care, we believe this trend points towards a broader acceptance of intensive care for patients with advanced pancreatic cancer given the recently seen improvements in systemic treatment.

However, the use of intensive care for patients with advanced pancreas in not without harm. It might increase overall costs and work load for health care professionals [16], rendering this strategy not cost-effective, especially in the setting of a public health system. Moreover, this approach might impose patients a more distressful end-of-life process. Therefore, it is crucial to understand the survival chances of patients with pancreatic cancer after an ICU admission and their ability to undergo further cancer-directed treatment. As far as the authors know, no previous study has evaluated exclusively the outcomes of patients with advanced pancreatic cancer admitted to the ICU due to clinical reasons. Earlier investigations with mixed populations with gastrointestinal malignancies (32%–36% of them with pancreatic cancer) have shown 30-day survival rates lower than 50% and 90-day survival rates ranging from 21% to 35% [17, 18]. Also, in these studies, nearly half of the patients died in the same hospital stay in which the ICU admission took place. Even though these studies do not provide data specifically for pancreatic cancer, its aggressive behaviour compared to other gastrointestinal malignancies suggest that these patients’ survival figures are even worse. Indeed, in a recent British survey, pancreatic cancer patients (any disease stage) with unplanned admissions to the ICU had the worst survival figures among all tumour types [19]. Despite the small number of patients with pancreatic cancer (*N* = 8), only 24% were alive at 30 days and 10.6% at 90 days. In our study, median overall survival was only 31 days, and 32% of patients were alive 90 days after the ICU admission. These data show us that while most patients with advanced pancreatic cancer who are admitted to the ICU do not benefit from intensive care, a significant minority might get the most out of it.

In this sense, it is important to identify those patients with advanced pancreatic cancer who are more likely to benefit from intensive care. With that in mind, we sat out to establish factors associated with higher chances of 30-day survival among these patients at the time of the ICU admission. While the choice of the 30-day landmark might seem arbitrary, previous data with ICU admission and other oncological interventions such as chemotherapy have used this timeline to evaluate futility in the setting of advanced cancer care [20, 21]. In our study, sepsis was the main cause for ICU admissions (49.7%). This is in line with data from recent studies that show that 35%–39% of the patients with advanced gastrointestinal malignancies are admitted to the ICU due to infectious complications [17, 18]. However, we could not find a clear relationship between cause of admission and survival. In this sense, we showed that ECOG performance status 0–1 was independently associated with higher odds of surviving 30 days or more. Despite not reaching statistical significance, other variables such as stage and treatment setting also were important in the logistic regression model. In one of the studies evaluating outcomes of patients with gastrointestinal malignancies admitted to the ICU due to clinical reasons, the authors also identified treatment setting as one of the most important factors affecting survival [17]. However, one should note that only 36% of the patients had pancreatic cancer and the other two factors associated with survival might suffer from overfitting (median time of metastatic disease) and lack of clinical usability by medical oncologists (Sequential Organ Failure Assessment [SOFA] score). Despite the significant amount of data correlating the use of physiological scales with survival in patients with cancer admitted to the ICU [22], we deliberately chose not to include such variables in our model as they add complexity and are not commonly used by medical oncologists, who are generally responsible to decide whether or not patients should be candidate to intensive care in our setting.

However, it might be difficult for patients and relatives to accept exclusive palliation in cases of advanced cancer, at least partially due to overly optimistic impressions about their survival [23]. Therefore, even the use of such tools to stratify patients’ chances of clinical benefit from the ICU might be hindered by the believe that a given patient might behave as an outlier. In this sense, it has been shown that patients with advanced pancreatic cancer that had early consultation with a palliative care specialist have lower rates of ICU admission toward the end-of-life [24, 25]. As a matter of fact, early palliative care has other potential advantages, such as improved quality of life for patients with pancreatic cancer [26] and even overall survival for patients with other solid tumours [27]. In our study, we could not collect data on the previous palliative care and quality of life in these patients, but we admit this issue merits further research.

Alternatively, one strategy that has been recently strengthened is the ICU trial approach [28]. In this situation, a patient is admitted to the ICU and limited medical interventions are given to select patients who respond to these interventions. Recent data suggest that for patients with poor-prognostic solid tumours (including 5%–9% of patients with pancreatic cancer), ICU trials lasting from 1 to 4 days might be sufficient to determine those who can benefit from further intensive care [29]. In our study, many patients were treated with this approach, as only a minority received vasoactive drugs, haemodialysis or mechanical ventilation. However, for patients clearly not responding to initial measures, discharge form the ICU and assistance from a palliative care group should be implemented since some data indicate the quality of death of patients with cancer in the ICU might be inferior [30]. Importantly, death outside the ICU allows family members to be by the patient’s side during her or his death process, which is frequently not possible in the ICU. In this sense, in our study, nearly one quarter of the patients died in the ICU, and importantly, nine of these patients died in the ICU without receiving any organ-supportive therapies. Thus, we believe that even if such ICU trials are implemented, one should make sure that once patients demonstrate signs of lack of response to initial measures, they are discharged from the ICU to a palliative care ward to receive proper care beside family members.

One strong argument in advanced cancer not to transfer a patient to the ICU is the low probability of receiving further oncological therapies. In our study, we showed that 34% of the patients received chemotherapy after ICU discharge. However, only 13.5% experienced clinical benefit from such a treatment. In the study by Epstein *et al* [18], no patient who underwent additional anti-cancer therapy appeared to benefit from it. This might be related to the relative chemo-resistance of pancreatic cancer, especially in more advanced stages. Not surprisingly, the factors associated with receiving further chemotherapy in our study parallel those associated with 30-day survival, which strengthens the 30-day survival outcome as an important one in this setting. Additionally, recent studies on the molecular biology of pancreatic cancer have shed light on specific subgroups of patients with increased response to platinum-based chemotherapy [31], poly(adenosine diphosphate-ribose) (poly[ADP-ribose]) polymerase inhibitors [32] and immune checkpoint inhibitors [33]. Perhaps, upfront knowledge of this information might change the medical oncologist’s drive to send patients to the ICU in the hope that such patients will eventually receive these treatments. However, only a minority of patients with pancreatic cancer currently have clearly targetable alterations, and this information is very frequently lacking before or during first-line treatment, when these therapies are most likely to be effective. Therefore, we believe our data still hold true until further developments in target therapy in pancreatic cancer are attained.

We acknowledge that our model has some limitations. Given the number of deaths at 30 days and the sample size, we had to reduce the number of degrees of freedom of the predictive variables. By doing that, we were able to comply with the ten events per degree of freedom rule, but we might have left behind important variables. Patients admitted to the ICU with acute respiratory failure had a very short overall survival, and this data is backed by the poor outcomes of patients undergoing mechanical ventilation. This is supported by previous data demonstrating poor survival for cancer patients who needed mechanical ventilation [34]. Also, model diagnostics demonstrate moderate discrimination capacity (c statistic of approximately 0.70) and suboptimal calibration. This might be partially related to the relatively small sample size [35], heterogeneity in intensive support for these patients and impossibility to add to the model potentially important variables, such as cause of ICU admission. Pending further external validation, however, we believe it can be used in conjunction with clinical reasoning to describe to the patients and their families the chances of benefiting from an ICU admission. Nonetheless our study has virtues. To the best of authors’ knowledge, this is the first study to portray the short-term prognosis of patients with advanced pancreatic cancer admitted to the ICU due to clinical reasons. Also, we provide data to guide treatment decisions regarding patients’ benefit from an ICU admission. We also reinforce the idea that clinical benefit from additional chemotherapy in this setting is uncommon. Finally, we provide a broad description of our model’s performance.

## Conclusion

To conclude, patients with advanced pancreatic cancer admitted to the ICU due to clinical reasons have poor prognosis. A minority of patients seem to benefit from admission to the ICU, especially those with previously preserved ECOG performance status, with stage III disease and in earlier phases of treatment. Also, only a small fraction will benefit from further oncological treatment. Further research is needed to consolidate our findings as they will help provide better care in the end-of-life for most of these patients and identify those who can benefit from intensive care.

## Statements and declarations

### Funding source

This study had no funding source.

### Conflicts of interest

None.

### Availability of data and material

The study database (as .csv file) can be obtained from the authors upon reasonable request.

### Code availability

The Stata codes (as .do file) for the statistical analyses can be obtained from the authors upon reasonable request.

### Author contributions

Marina Junqueira de Almeida: conceptualisation, data curation, writing and visualisation; Marcos Pedro Guedes Camandaroba: data curation, writing and visualisation; Antonio Paulo Nassar Jr: conceptualisation, methodology and visualisation; Victor Hugo Fonseca de Jesus: conceptualisation, methodology, data analysis, writing and visualisation.

### Ethics approval

This study was approved by the Institutional Ethics Board Review, which waived the requirement for informed consent due to the retrospective and observational design of the study (number 2521/18N).

### Consent to participate

The Institutional Ethics Board Review waived the requirement for informed consent due to the retrospective and observational design of the study.

### Consent to publish

Not applicable.

## Figures and Tables

**Supplementary Table 1.** Overall survival according to reason for ICU admission (*N* = 171).

Reason	*N*	Median overall survival – days (95% CI)	*p*
Sepsis	85	32 (19–49)	0.452
Vascular event	22	19 (10–55)	
Acute respiratory failure	20	17 (7–71)	
Gastrointestinal bleeding	20	66 (12–136)	
Others	24	64 (6–179)	

**Supplementary Table 2.** Overall survival according to the use of specific organ-supportive therapies (*N* = 171).

Therapy	*N*	Median overall survival – days (95% CI)	*p*
Vasoactive drugs – *N* (%) Yes No	69 102	24 (13–49) 37 (24–65)	0.147
Haemodialysis – *N* (%) Yes No	5 166	14 (5–NA) 33 (23–54)	0.018
Mechanical ventilation – *N* (%) Yes No	31 140	11 (5–19) 39 (28–66)	< 0.001

**Supplementary Table 3.** Model diagnostics.

Metric	Apparent performance	Optimism-corrected performance
Link test *p*-value	0.624	-
Hosmer–Lemeshow *p*-value	0.776	-
Scaled Brier score	0.122	0.074
c statistic	0.697	0.672

**Supplementary Table 4.** Optimism-adjusted multivariate regression model.

	Multivariate analysis	
Variable	OR (95% CI)	*p*
Age	1.02 (0.99–1.04)	0.285
ECOG performance status ECOG 0–1 ECOG 2–4	1.00 0.33 (0.18–0.58)	< 0.001
Stage III IV	1.00 0.45 (0.19–1.06)	0.068
Treatment setting Before or during first-line During second- or further line or BSC	1.00 0.71 (0.39–1.32)	0.281

OR, Odds ratio; 95%CI, 95% confidence interval; ECOG, Eastern Cooperative Oncology Group; BSC, Best supportive care

**Supplementary Table 5.** Logistic regression model for receiving additional systemic chemotherapy (*N* = 162 for multivariate analysis).

	Univariate analysis		Multivariate analysis	
Variable	OR (95% CI)	*p*	OR (95% CI)	*p*
Age	1.01 (0.98–1.04)	0.679		
Sex Male Female	1.00 1.40 (0.74–2.68)	0.299	1.00 1.51 (0.74–3.07)	0.253
ECOG performance status ECOG 0–1 ECOG 2–4	1.00 0.35 (0.18–0.67)	0.002	1.00 0.28 (0.13–0.57)	< 0.001
Charlson comorbidity score	1.14 (0.80–1.63)	0.460	1.38 (0.92–2.08)	0.119
Stage III IV	1.00 0.45 (0.19–1.06)	0.067	1.00 0.47 (0.19–1.19)	0.110
Treatment setting Before or during first-line During second- or further line or BSC	1.00 0.55 (0.27–1.13)	0.105	1.00 0.59 (0.27–1.29)	0.188

OR, Odds ratio; 95%CI, 95% confidence interval; ECOG, Eastern Cooperative Oncology Group; BSC, Best supportive care

**Table 1.** Demographic and clinical characteristics of the study population.

Characteristic	Study population (*N* = 171)
Age – years Median (range)	66 (39–87)
Period – *N* (%) 2009–2012 2013–2015 2016–2018	17 (9.9) 71 (41.5) 83 (48.5)
Sex – *N* (%) Male Female	87 (50.9) 84 (49.1)
ECOG performance status – *N* (%) ECOG 0 ECOG 1 ECOG 2 ECOG 3 ECOG 4	10 (5.9) 67 (39.2) 87 (50.9) 6 (3.5) 1 (0.6)
Age-adjusted Charlson comorbidity index Median (IQR)	9 (7–10)
Stage – *N* (%) III IV	25 (14.6) 146 (85.4)
Number of metastatic sites Median (IQR)	1 (1–2)
Hepatic metastasis – *N* (%) Yes No	104 (60.8) 67 (39.2)
Treatment setting Before first-line First-line Second-line Third- or fourth-line BSC Unknown	40 (23.4) 75 (43.9) 34 (19.9) 19 (11.1) 2 (1.2) 1 (0.6)

ECOG, Eastern Cooperative Oncology Group; IQR, Interquartile range

**Table 2.** Reasons for the ICU admission (*N* = 171).

Group of reasons for admission	*N* (%)	Reason for admission	*N* (%)
Sepsis	85 (49.7)	Biliary sepsis Other sites of sepsis	39 (22.8) 46 (26.9)
Cardio/cerebrovascular event	22 (12.9)	Cardio/cerebrovascular event	22 (12.9)
Acute respiratory failure	20 (11.7)	Acute respiratory failure	20 (11.7)
Gastrointestinal bleeding	20 (11.7)	Gastrointestinal bleeding	20 (11.7)
Other reasons	24 (14.0)	Encephalopathy Acute renal injury Treatment toxicity Cranioencephalic trauma Shock from unknown cause Hepatic failure Acute pancreatitis Other causes	7 (4.1) 4 (2.3) 4 (2.3) 2 (1.2) 2 (1.2) 1 (0.6) 1 (0.6) 3 (1.8)

**Table 3.** Final logistic regression model for 30-days survival (*N* = 167 for multivariate analysis).

	Univariate analysis		Multivariate analysis	
Variable	OR (95% CI)	*p*	OR (95% CI)	*p*
Age	1.01 (0.99–1.05)	0.330	1.02 (0.99–1.05)	0.285
Sex Male Female	1.00 1.05 (0.57–1.93)	0.877		
ECOG performance status ECOG 0–1 ECOG 2–4	1.00 0.29 (0.15–0.56)	< 0.001	1.00 0.28 (0.14–0.54)	< 0.001
Charlson comorbidity score	0.93 (0.66–1.32)	0.701		
Stage III IV	1.00 0.37 (0.15–0.95)	0.038	1.00 0.40 (0.15–1.07)	0.068
Treatment setting Before or during first-line During second- or further line or BSC	1.00 0.70 (0.36–1.33)	0.274	1.00 0.68 (0.34–1.37)	0.281

OR, Odds ratio; 95% CI, 95% confidence interval; ECOG, Eastern Cooperative Oncology Group; BSC, Best supportive care
